# Adapting measures of motor imitation for use by caregivers in virtual contexts: Reliability, validity, and sensitivity to change

**DOI:** 10.1002/aur.3267

**Published:** 2024-11-21

**Authors:** Brooke Ingersoll, Mya Howard, Devon Oosting, Alice S. Carter, Wendy L. Stone, Natalie Berger, Allison L. Wainer, Emily R. Britsch, RISE Research Network, Sarabeth Broder‐Fingert, Alice Carter, Sarah Edmunds, Brooke Ingersoll, Chris Sheldrick, Wendy Stone, Allison Wainer

**Affiliations:** ^1^ Department of Psychology Michigan State University East Lansing Michigan USA; ^2^ Department of Psychology University of Massachusetts Boston Boston Massachusetts USA; ^3^ Department of Psychology University of Washington Seattle Washington USA; ^4^ Autism Assessment, Research, and Treatment Center, Department of Psychiatry and Behavioral Sciences Rush University Medical Center Chicago Illinois USA

**Keywords:** assessment, autism, caregiver‐implemented, imitation, virtual

## Abstract

Early imitation challenges for children with autism are thought to contribute to broader delays in their social communication development. As such, imitation is an important intervention target for young children with and showing early signs of autism, and efforts are underway to disseminate evidence‐based imitation interventions into community settings. To our knowledge, there are currently no established imitation assessments that have been validated for use in virtual contexts. This study was designed to examine the reliability and validity of two caregiver‐implemented imitation measures delivered with support from a remote virtual assessor. Study participants (177 caregiver‐child dyads) were enrolled in a large, multisite study that is examining the effectiveness of a caregiver‐implemented intervention delivered through the Part C early intervention (EI) system across four states. Results indicate that the assessments can be administered remotely with strong fidelity, internal reliability, predictive validity, discriminant validity, convergent validity, and sensitivity to change. Stability over time was adequate. These findings suggest that imitation skills can be measured effectively using a remote caregiver‐implemented assessment, which provides greater opportunity for virtual clinical trials targeting social communication in young children.

**Clinical Trial Registration:**

The trial protocol was registered at ClinicalTrials.gov (NCT05114538).

## Lay Summary

Imitation is an important skill for young children because it is an aspect of social communication development. Early intervention can increase imitation skills in young children with social communication delays, including autism. Therefore, it is important to have tools that can measure imitation skills and track imitation progress during interventions, and that can reach a broad population. Currently, there are no available tools to do so. This study explores whether two virtual, caregiver‐implemented imitation assessments measure what they are supposed to measure and do so consistently. Study participants (177 caregivers and their child) were enrolled in a large, multisite study that is examining the effectiveness of a caregiver‐implemented intervention delivered through the Part C early intervention (EI) system in four states. Results show that the virtual assessments can be administered correctly, consistently measure what they are supposed to measure, and can detect changes in imitation skills over time. These findings suggest that imitation skills can be measured effectively using virtual caregiver‐implemented assessments, which could help improve research and clinical interventions targeting imitation skills in young children.

## INTRODUCTION

Imitation skills emerge within the first year of life and serve an important role in early social communication development, by facilitating the acquisition of language and reciprocal interactions (Bates et al., 1979; Meltzoff, 2005; Nieto & Campos, 2022). Autistic children show delays in imitation skills; they imitate less often and are less precise in their imitation than their non‐autistic peers (Edwards, 2014; Williams et al., 2004). It has been proposed that early imitation difficulties in autism lead to broader delays in social communication development, based on the central role that imitation plays in learning (Rogers & Pennington, 1991). In support of this theory, studies have found imitation skills in young autistic children to be associated with language, both concurrently and over time (Frost et al., 2024; Pittet et al., 2022; Stone & Yoder, 2001; Toth et al., 2006). Despite imitation challenges, autistic children may rely on imitation more than joint attention to learn language (Carpenter et al., 2002), and there is some evidence that improving imitation skills may lead to broader improvements in other social communication skills (Ingersoll, 2012; Ingersoll & Schreibman, 2006; Yoder et al., 2021). These findings make imitation an important target of EI (Sandbank et al., 2017).

In young children, imitation is usually assessed via live administration of an assessment battery by a trained examiner. Commonly used measures include the Motor Imitation Scale (MIS; Stone et al., 1997), the Preschool Imitation and Praxis Scale (PIPS; Vanvuchelen et al., 2011), the visuo‐Motor Imitation Scale of the Psychoeducational Profile, 3rd Edition (PEP‐3; Schopler et al., 2005), Roger's imitation battery (Rogers et al., 2003), and the Unstructured Imitation Assessment (UIA; Ingersoll & Meyer, 2011a). Most assessments include imitation of single‐step actions with objects and body/gesture imitation, and include both meaningful and non‐meaningful actions. The majority of assessments focus on structured‐elicited imitation (i.e., child and examiner seated at a table and the child is given explicit instruction to imitate). However, some involve spontaneous elicitation of imitation within a social interaction (i.e., examiner imitates the child and then presents models without explicit instructions to imitate). Studies have shown that autistic children typically have better imitation with objects than gestures (Ingersoll & Meyer, 2011b; Stone et al., 1997), better imitation of meaningful than non‐meaningful actions (Stone et al., 1997; Williams et al., 2004), and better structured‐elicited than spontaneous‐social imitation (Ingersoll & Meyer, 2011a).

Given the recent increase in virtually conducted clinical trials (Cummings, 2021; Ng et al., 2023), as well as telehealth‐delivered clinical care (Ellison et al., 2021), it is important to develop methods to elicit these skills remotely in the home environment (Ozonoff et al., 2024). For young children, the most ecologically valid approach to elicit skills virtually involves caregiver administration (e.g., BOSA, Tele‐ASD‐PEDS; Dow et al., 2022; Wagner et al., 2021). There is growing research showing that remote caregiver assessment can be used effectively for autism screening and diagnosis (Berger et al., 2022) as well as for functional analysis of behavior (Schieltz & Wacker, 2020). However, these studies have focused on making clinical determinations about autism, developmental level, or the function of child behaviors. In contrast, outcome measures require reliable assessment of individual differences as well as change over time for a specific construct. Little is known about the validity of using caregiver‐implemented assessment to measure explicit imitative behaviors. In an increasingly telehealth‐friendly age, it is necessary to validate tools to measure relevant social communication skills in young children that can be administered by caregivers in their own homes.

We developed a caregiver‐implemented imitation assessment protocol for use in a hybrid effectiveness‐implementation type 1 trial of an imitation‐based intervention, Reciprocal Imitation Teaching (RIT) in the Part C EI system. The goal was to have the caregiver administer the assessment at home with the support of a remote virtual assessor. Toward this end, we used user‐centered design principles to modify two examiner‐administered measures of imitation that have been used successfully in studies with young autistic children (MIS and UIA) for use by caregivers.^1^ Modifications involved: (1) reducing the number of items to make administration more feasible; (2) developing clear procedures and scripts to support caregiver administration; (3) conducting pilot testing with several families; (4) and assessing feasibility and acceptability with families and research staff with varying backgrounds. The objective of the current study is to evaluate the reliability and validity of the resulting caregiver‐implemented imitation measures.

## METHOD

### Participants

The sample for the present study comprised 177 caregiver‐child dyads. All participants were enrolled in a large, multisite, federally funded study that is examining the effectiveness of a caregiver‐implemented intervention delivered through the Part C EI system across four states (NCT05114538). Data collection is still ongoing. For this study, EI providers were randomly assigned to either receive training in caregiver‐implemented RIT (RIT‐Now) or to provide usual care, with the option of receiving RIT training at the end of the study (RIT‐Later). Data from children in both groups were included in this study.

Children were initially referred to the study by their EI providers based on concerns about their social communication development (i.e., early signs of autism). Eligibility criteria included child age between 16 and 30 months at enrollment, family speaking either Spanish or English, and having sessions with their EI provider at least once per week during their 4‐month, active treatment phase. Families were excluded if their child had significant motor, hearing, or visual impairments that would impede their ability to complete the activities. The sample was diverse and representative of young children with early signs of autism in the Part C EI system for the four states (see Table 1 for sociodemographic characteristics).

**TABLE 1:** Participant demographics.

	Mean (SD)/frequency (percentage)
Child demographics (*n* = 177)	
Age (months)	26.55 (3.81)
Sex (% male)	119 (67.2%)
Race	
Indigenous/Native Alaskan	1 (0.6%)
Asian	9 (5.1%)
Native Hawaiian/Pacific Islander	0 (0%)
Black/African American	34 (19.2%)
White	95 (53.7%)
Other	4 (2.3%)
More than one race	22 (12.4%)
Prefer not to answer	12 (6.8%)
Race/ethnicity (% minoritized status)	102 (61.4%)
PIA‐CV motor imitation	10.33 (3.90)
VABS Expressive Language (GSV)	88.72 (17.38)
VABS Receptive Language (GSV)	70.36 (18.83)
MacArthur‐Bates CDI (Total Words Said)	15.10 (24.54)
Caregiver demographics (*n* = 177)	
Age (years)	33.76 (6.23)
Gender (% woman)	158 (89.3%)
Race/Ethnicity (% minoritized status)	99 (59.6%)
Education (% college degree)	78 (44.6%)
Marital status (% living with partner)	138 (78.0%)
Household income	
Less than $10,000	15 (9.6%)
$10,000–$24,999	16 (10.3%)
$25,000–$49,999	38 (25.0%)
$50,000–$74,999	21 (9.6%)
$75,000–$99,999	15 (9.0%)
$100,000–$149,999	14 (7.7%)
$150,000–$174,999	6 (3.8%)
$175,000–$199,999	3 (1.9%)
$200,000 or above	15 (9.6%)

Abbreviation: GSVs, Growth Scale Values.

### Procedure

Caregiver‐child dyads were assessed at baseline (T1), after 4 months of intervention (T2), and after a 5‐month follow‐up (T3) using the RISE Communication Play Protocol (CPP). All caregivers were naïve to the virtual caregiver‐implemented assessment of child social communication skills at the T1 assessment point. The CPP is a caregiver‐implemented assessment of social communication skills that was adapted from the work of Lauren Adamson and Roger Bakeman (Adamson & Bakeman, 2016). Specific activities assessed children's motor imitation, joint attention, object play, and intentional communication. Activities were presented in a playful context. Caregivers were coached and supervised through a videoconferencing platform (Zoom) by trained research staff blind to the intervention status of the family. A technology kit, including cameras and Wi‐Fi, as well as a standard set of toys were provided to the families for use during the assessment (Petruccelli et al., 2024; Tagavi et al., 2024). At each time point, caregivers completed online surveys through REDCap (Harris et al., 2009). Study procedures were approved by the Single Institutional Review Board for the multisite trial at Michigan State University. Written informed consent was collected from caregivers, as well as EI providers, prior to participating in the study.

### Measures

#### Demographic survey

Caregivers completed a survey assessing sociodemographic characteristics, including caregiver and child age, sex, gender, race, ethnicity, as well as caregiver educational level, income level, and marital status (lives with spouse or partner). This measure was used to characterize the sample and analyze discriminant validity. For correlational analyses, race and ethnicity were combined and dichotomized into minorized status (Black/African American, Asian, Indigenous/Native Alaskan, Native Hawaiian/Pacific Islander, More than One Race, Hispanic or Latinx, Other) and non‐minoritized status (White, non‐Hispanic or Latinx). Caregiver educational level was dichotomized into less than college degree and college degree or higher.

#### Caregiver‐implemented assessments of imitation

Trained virtual assessors coached caregivers to administer two imitation assessments to their child over Zoom as part of the RISE CPP. Prior to each imitation assessment, the virtual assessor provided verbal and written instructions to the caregiver (see Table 2). The virtual assessor first demonstrated each action to be modeled with a duplicate of the toys from the assessment kit, and then asked the caregiver to model the action for the child, providing additional prompts or support as needed. As the child was present during the assessment, the child was able to hear the virtual assessor's prompts at the same time as the caregiver. The child's response to each model from the caregiver was scored live by the virtual assessor. All assessments were recorded for fidelity monitoring and reliability coding. A description of the actions modeled can be found in Table 3.

**TABLE 2:** Example instructions for *You Do It* and *Copy Cat.*

Caregiver Cue card	Virtual assessor instructions script
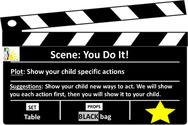	For the *You Do It* scene, I will show you an action and a sound, and ask you to do and say the same thing to your child. After you show your child the action, try to get them to do it by saying “You do it.” I may have you repeat the action a few times.
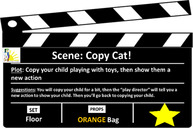	This scene is called *Copy Cat*…In this scene, you'll take turns copying [Child's name] and giving him/her the chance to copy you. Some children love this copy game, while others do not show much interest in it. No matter how your child responds, it is absolutely ok! To start, your job is to watch what [Child name] does with the toys and copy him/her. We will do that for about a minute. Then I'm going to ask you to show [Child name] a new way to play with one of the toys. We will switch back and forth between copying your child and showing him/her new ways to play for about 10 min. One thing to remember here—try not to give your child specific instructions to copy you. So please do not say “You Do It” or “Your Turn!” In this scene, we just want to see how your child reacts to you copying everything they are doing, and also how [Child Name] responds to you doing something new with some toys. I'm going to leave my camera on so that when it's time to show your child a new action, I can show you the action on the screen.

**TABLE 3:** Item Analysis for You Do It and Copy Cat.

	M (SD)	Fail (0)	Emerging (1)	Pass (2)	Corrected item‐total correlation	Cronbach's *α* if item removed
You Do It (*N* = 166)	Range 0–2					*α* = 0.63
Drum hands on table	0.46 (0.74)	68%	16%	16%	0.28	0.63
Place block on head	0.63 (0.84)	61%	15%	23%	0.40	0.57
Hop dog across tabletop	0.64 (0.81)	55%	23%	22%	0.52	0.51
Push car across tabletop	1.27 (0.87)	27%	19%	54%	0.44	0.55
Shake rattle	1.49 (0.85)	23%	5%	72%	0.29	0.62
Copy Cat (*N* = 162)	Range 0–2					*α* = 0.70
Feed animal food	0.33 (0.71)	81%	5%	14%	0.44	0.66
Roll ball back and forth on tabletop 3X	0.60 (0.87)	64%	8%	28%	0.40	0.67
Drop fish in the net	0.74 (0.87)	53%	18%	29%	0.49	0.64
Turn sound tube upside down 2X	0.74 (0.83)	50%	24%	26%	0.29	0.70
Roll car off upside down container	0.75 (0.93)	59%	7%	33%	0.42	0.66
Hit tambourine with maraca	0.76 (0.92)	57%	11%	32%	0.53	0.62

*You Do It* (YDI) was adapted from the (MIS; Stone et al., 1997) to evaluate structured‐elicited imitation. A total of five imitation tasks (four involving actions with objects and one involving a body movement) were selected from the original scale based on suitability for this age range. The caregiver was asked to obtain the child's attention and then model the specific action. After the model, the caregiver placed the toy in front of the child and gave the instruction, “Your turn, you do it.” The child was given a 5‐s response period after each trial, and up to 3 trials could be given for each item. Each trial was scored on a 3‐point scale, with a score of “2” indicating that the child produced an exact imitation, “1” indicating an emerging imitation (i.e., the child partially imitated the action but failed to do so in the exact manner it was presented), and “0” indicating that the child failed to imitate the model. The child's best response for each action was used to calculate the overall score, which could range from 0 to 10.

*Copy Cat* (CC) was adapted from the (UIA; Ingersoll & Meyer, 2011a) to measure spontaneous‐social imitation. Caregiver‐child dyads were seated on the floor with 2 duplicate sets of 6 toys freely available. The caregiver was asked to begin the assessment by imitating everything the child did (i.e., imitating all child vocalizations, movements, and actions on objects) for 45 s (i.e., contingent imitation). If the child manipulated a toy during this period, this action was imitated by the parent using the available duplicate toy. After the contingent imitation period, the caregiver was asked to place a toy in front of the child and then model an action and accompanying verbal marker with the duplicate toy without giving explicit directions to imitate (e.g., caregiver makes animal toy pretend to drink from a cup and says, “Animal is thirsty! Mmm”). The child was given a 5‐s response period after each trial. The caregiver was prompted by the virtual assessor to model the action up to three times, depending on the child's response, and then return to imitating their child for 45 s. Models could be presented in any order, but the virtual assessor refrained from asking the caregiver to model an action with a toy the child was already holding. Trials were scored on a 3‐point scale, with a score of “2” indicating that the child fully imitated the caregiver's actions, a “1” indicating an emerging response, and a “0” indicating that the child did not imitate. The child's best response for each action was used for the overall score, which could range from 0 to 12. A description of the actions modeled can be found in Table 3.

##### Virtual assessor training

Assessment training for the virtual assessors included didactics, role plays, and at least three supported assessment administrations. Once a new virtual assessor reached fidelity on both administration and live scoring, they could administer assessments independently.

##### Assessment fidelity

Assessment fidelity for virtual assessors and caregivers was scored from video by trained coders for 20% of administrations using assessment fidelity checklists, which specified procedures for each measure. Virtual assessor fidelity was calculated as the percent of coaching procedures with the caregiver that were implemented correctly by the assessor for each imitation measure. Caregiver fidelity was calculated by scoring the caregiver on correct implementation of each item with their child and taking the percent of items implemented correctly for each imitation measure.

##### Interrater reliability

The children's performance on each imitation measure was scored from video by a trained coder for 20% of administrations, and an intraclass correlation coefficient (ICC) was used to determine interrater reliability.

#### Caregiver measures

##### Parent interview for autism‐clinical version

The parent interview for autism‐clinical version (PIA‐CV; Stone et al., 2003) is a caregiver‐report measure of autism symptom severity in young children. Caregivers rate each item's typical frequency of occurrence for their child ranging from 1 “*almost never*” to 5 “*almost always*.” The total score for the 4‐item Motor Imitation domain (*α* = 0.73) was used in the current study to examine convergent validity.

##### Vineland adaptive behavior scales‐third edition, comprehensive interview form

The Vineland adaptive behavior scales‐third edition (VABS‐3; Sparrow et al., 2016) is a well‐validated, structured interview that assesses four domains of adaptive skills: communication, socialization, daily living skills, and motor skills. All items are rated on their frequency of occurrence on a scale ranging from 0 (*never*) to 2 (*usually*). For the current study, Growth Scale Values (GSVs) for the Expressive Language and Receptive Language subdomains were used to evaluate convergent and predictive validity.

##### MacArthur‐Bates communicative development inventory short form vocabulary checklist

The MacArthur‐Bates communicative development inventory (MB‐CDI; Fenson et al., 2000) words and sentences short form vocabulary checklist is a 100‐word checklist that measures early Expressive Language development in children 16–30 months of age. Caregivers indicate which words on the checklist their child “says on his/her own.” The total raw score for words generated (Total Words Said) was used to evaluate convergent and predictive validity.

##### Brief infant‐toddler social and emotional assessment

The brief infant‐toddler social and emotional assessment (BITSEA; Briggs‐Gowan et al., 2004) is a well‐validated measure of early social–emotional behavioral problems in children aged 12–36 months. Caregivers complete 42 items that are rated on a 3‐point scale (0 = *not true/rarely*, 1 = *somewhat true/sometimes*, 2 = *very true/often*). There are also two items in which caregivers report their concerns about their child's behavior and language development from 1 to 5, with higher scores indicating greater concern. The BITSEA yields two scales: problem and competency. The BITSEA Problem Scale was used in the current study to assess discriminant validity.

## RESULTS

Virtual assessor and caregiver fidelity were high across the 20% of administrations in which they were examined. Average virtual assessor fidelity was 94% (Range 67%–100%) for YDI and 94% (Range 75%–100%) for CC. Average caregiver fidelity was 97% (Range 60%–100%) for YDI and 94% (Range 34%–100%) for CC.

We examined the mean, standard deviation (SD), and percent of children failing, emerging, or passing individual items for YDI and CC at T1 for the full sample. Imitation items on YDI and CC represented a range of difficulty with children passing between 16% and 72% of items on YDI and between 14% and 33% on CC.

Baseline YDI and CC total scores for the full sample were examined for skewness and kurtosis and were found to be normally distributed. Cronbach's alpha coefficients were computed for each imitation measure to examine internal consistency. For YDI, *α* = 0.63 and the corrected item‐total correlations ranged from 0.28–0.52. For CC, *α* = 0.70 and corrected item‐total correlations ranged from 0.29 to 0.53. No items, if removed, would have improved the reliability of either measure, indicating adequate to good internal consistency for YDI and CC, respectively (see Table 3).

As previous research has established that structured‐elicited imitation is more frequent in young autistic children than spontaneous‐social imitation (Ingersoll & Meyer, 2011a), we compared patterns of imitation performance on YDI and CC at T1 (for the full sample) and T2 (for the RIT later sample) using paired‐samples *t*‐tests to assess whether this same pattern holds for the remote protocol. To ensure comparability between measures, we converted raw scores for both measures to percent correct by dividing each child's overall score by the maximum possible score for that measure. Children performed significantly better on YDI (*M* = 44.62, *SD* = 25.89) than CC (*M* = 32.42, *SD* = 26.71) at T1, *t*(172) = 5.58, *p* < 0.001, as well as at T2 (YDI: *M* = 54.84, *SD* = 30.55; CC: *M* = 42.84, *SD* = 33.30), *t*(63) = 2.94, *p* = 0.005, consistent with previous findings using the MIS and UIA (Ingersoll & Meyer, 2011a).

Interrater reliability of child imitation performance was calculated for 20% of assessment administrations using ICC. The ICC values obtained indicate excellent interrater reliability for YDI (ICC = 0.95) and CC (ICC = 0.94). Four‐month stability was examined in the RIT later group only,^2^ by examining the correlation between imitation performance on T1 and T2 assessment administrations (Briggs‐Gowan et al., 2004). For both measures, Pearson's r values were fair (YDI: *r* = 0.44, CC: *r* = 0.54), indicating moderate stability in imitation performance on these measures.

Convergent validity was examined in the full T1 sample using Pearson's product moment correlations between YDI, CC, and a caregiver‐report measure of imitation (PIA‐CV Motor Imitation subscale) as well as with three measures of child language (GSV scores from the Expressive Language and Receptive Language subdomains on the VABS‐3, and Total Words Said on the MB‐CDI). As expected, YDI and CC were positively correlated with each other (*r*(171) = 0.40, *p* < 0.01), and both measures were positively correlated with the PIA‐CV Motor Imitation subscale (YDI: *r*(173) = 0.31, *p* < 0.01; CC: *r*(171) = 0.53, *p* < 0.01). In addition, both YDI and CC were moderately correlated with each of the language measures, suggesting convergent validity (see Table 4). Fisher's *r*‐to‐*z* transformation was used to compare whether the correlations between YDI and CC and the convergent validity measures were statistically significant. There was a statistically significant difference in correlations between observed YDI and CC and the caregiver‐reported Motor Imitation subscale on the PIA‐CV, *z* = −2.49, *p* = 0.01, indicating that CC was more strongly correlated with the PIA‐CV Motor Imitation subscale than YDI. There were no other significant differences in correlations between YDI and CC and associated measures.

**TABLE 4:** Pearson's r correlations between measures.

	You Do It	Copy Cat
Convergent validity (*N* = 177)		
You Do It (% correct)	‐	0.40**
Copy Cat (% correct)	0.40**	‐
PIA‐CA motor imitation	0.31**	0.53**
VABS Expressive Language (GSV)	0.24**	0.39**
VABS Receptive Language (GSV)	0.30**	0.42**
MacArthur‐Bates CDI (Total Words Said)	0.17*	0.30**
Discriminant validity (*N* = 177)		
Child age (months)	−0.02	0.04
Child sex	0.09	0.00
Child race/ethnicity (% minoritized status)^b^	−0.02	0.05
Child total problems (BITSEA)	−0.03	−0.04
Caregiver age (years)	0.06	0.01
Caregiver race/ethnicity (% minoritized status)^b^	−0.08	−0.01
Caregiver marital status (% living with partner)	−0.02	−0.10
Caregiver education (% college degree)	0.03	0.01
Family income^a^	0.02	−0.11

Abbreviation: GSVs, Growth Scale Values.

^a^ Spearman's rho.

^b^ Non‐minoritized = White, non‐Hispanic.

* *p* < 0.05.

** *p* < 0.01.

Discriminant validity was examined by correlating YDI and CC in the full T1 sample with child demographics (age, sex, race/ethnicity) and caregiver demographics (age, gender, race/ethnicity, marital status, education, family income), as well as a measure of child behavior problems (BITSEA Total Problems Scale), which has not previously been associated with imitation. YDI and CC did not significantly correlate with any child or caregiver demographics (all *r*s <0.12, all *p*s >0.05). Further, neither imitation measure was correlated with the BITSEA Total Problem Scale, suggesting strong discriminant validity (see Table 4).

Predictive validity was examined with the subset of children in the RIT later group who had completed their T3 assessment (*n* = 55), using a series of hierarchical linear regressions to examine whether performance on the two imitation measures predicted language growth over 9 months on the three language measures (VABS‐3 Expressive Language GSV, VABS‐3 Receptive Language GSV, MB‐CDI Total Words Said). For each model, the relevant T3 language measure was entered as the outcome. The corresponding T1 language measure was entered in Step 1, and the T1 imitation measure (YDI or CC) was entered in Step 2 as predictors. In separate models, both YDI (*β* = 0.28, *t* = 3.27, *p* < 0.01) and CC (*β* = 0.26, *t* = 2.75, *p* < 0.01) were significant predictors of growth in Expressive Language on the VABS‐3 after controlling for T1 Expressive Language. In both models, the imitation measure explained an additional 7% of the variance in Expressive Language. Neither YDI or CC were significant predictors of growth in Receptive Language on the VABS‐3 or total words said on the MB‐CDI. See Tables 5 and 6.

**TABLE 5:** Hierarchical regression predicting Expressive Language growth over 9 months from T1 You Do It.

Variable (*N* = 55)	Model 1		Model 2	
	*β*	*t*	*β*	*t*
	VABS Expressive Language (GSV) at T3			
VABS Expressive Language (GSV) at T1	0.73	7.93**	0.70	8.13**
You Do It at T1			0.28	3.27**
Adjusted *R* ^*2*^	0.53		0.60	
*F* change	62.85**		10.72**	
	VABS Receptive Language (GSV) at T3			
VABS Receptive Language (GSV) at T1	0.62	0.58**	0.57	5.07**
You Do It at T1			0.15	1.32
Adjusted *R* ^*2*^	0.37		0.38	
*F* change	33.40**		1.75	
	MacArthur‐Bates CDI Total Words Said at T3			
MacArthur‐Bates CDI (Total Words Said) at T1	0.56	6.00**	0.54	5.63**
You Do It at T1			0.09	0.97
Adjusted *R* ^*2*^	0.31		0.31	
*F* change	35.91**		0.94	

Abbreviation: GSVs, Growth Scale Values.

** *p* < 0.01.

**TABLE 6:** Hierarchical regression predicting Expressive Language growth over 9 months from T1 Copy Cat.

Variable (*N* = 54)	Model 1		Model 2	
	*β*	*t*	*β*	*t*
	VABS Expressive Language (GSV) at T3			
VABS Expressive Language (GSV) at T1	0.72	7.65**	0.64	6.71**
Copy Cat at T1			0.26	2.75**
Adjusted *R* ^*2*^	0.52		0.59	
*F* change	58.50**		7.58**	
	VABS Receptive Language (GSV) at T3			
VABS Receptive Language (GSV) at T1	0.62	5.75**	0.55	4.67**
Copy Cat at T1			0.18	1.55
Adjusted *R* ^*2*^	0.37		0.41**	
*F* change	33.04		2.41	
	MacArthur‐Bates CDI Total Words Said at T3			
MacArthur‐Bates CDI (Total Words Said) at T1	0.56	5.87**	0.51	5.13**
Copy Cat at T1			0.18	
Adjusted *R* ^*2*^	0.31		0.33	
*F* change	34.48**		3.13	

Abbreviation: GSVs, Growth Scale Values.

** *p* < 0.01.

Lastly, in order to examine sensitivity to change for YDI and CC, we ran paired samples *t*‐tests comparing T1 to T2 performance on each measure for children in the RIT later group (*n* = 64). The children made significant improvements from T1 to T2 in YDI performance (T1: *M* = 46.40, SD = 25.41; T2: *M* = 54.84, SD = 30.55), *t*(63) = −2.26, *p* = 0.03, and CC performance (T1: *M* = 32.03, SD = 29.30; T2: *M* = 42.84, SD = 33.30), *t*(63) = −2.85, *p* = 0.006, suggesting that the measures are sensitive to change over time.

## DISCUSSION

The present study examined measurement characteristics of two adapted imitation assessments in young children displaying early signs of autism when implemented by the child's caregiver in the family home, with virtual assessor support. The YDI and CC measures were both able to be administered with a high degree of fidelity by virtual assessors and caregivers, as well as high interrater reliability, suggesting that virtual caregiver‐implemented assessment of imitation skills in young children with early signs of autism is feasible. In addition, item analysis suggested that the YDI and CC measures performed well. The corrected item‐total correlations for all YDI and CC items were greater than 0.25, with most between 0.30 and 0.70, and no items, if removed, would improve the reliability of the scales. In addition, no items on either measure were too hard (<10% pass) or too easy (<10% fail). CC demonstrated adequate internal validity (*α* = 0.70). While the internal validity of YDI was slightly low (*α* = 0.63), it was within the range of acceptability. Given that Cronbach's alpha is affected by item number within a scale, YDI may benefit from adding another item (for a total of 6 items), which would likely increase its internal validity and make it more comparable to CC which has 6 items.

The mean performance on both measures was higher than expected based on previous research with autistic children ages 2–4 years using the original measures (Ingersoll & Meyer, 2011a). This discrepancy seemed to be more pronounced for CC than YDI. Due to the young age of the children in the current study, we eliminated items from the MIS and UIA with the lowest pass rates, which may be why performance was higher. It is also possible that child performance was higher in this study because the measures were administered by the caregiver in a familiar setting (the home), rather than by an examiner in a novel setting (e.g., the lab). At the same time, child performance on YDI was significantly higher than on CC, which is consistent with previous research (Ingersoll & Meyer, 2011a), suggesting the caregiver‐implemented version may be performing similarly to the examiner‐administered version. Additional research is needed to determine if the method of administration affects child performance.

Correlations between two administrations of the imitation measures conducted 4 months apart were in the moderate range, suggesting that the children's relative performance is moderately stable, despite many children showing growth in these skills over time. This finding is interesting in light of different developmental patterns in imitation development that have been observed in young autistic children, with some showing significant improvement and others not (Pittet et al., 2022). A limitation to this work is that we did not examine test‐retest reliability over a short period of time (i.e., 1–2 weeks); future research that examines test‐retest reliability would be highly beneficial.

Validity analyses indicated that there was evidence of convergent validity, with the two imitation measures positively correlated with each other and with a caregiver‐reported measure of imitation skills. In addition, both imitation measures were positively correlated with concurrent measures of Expressive and Receptive Language skills, consistent with previous research (Ingersoll & Meyer, 2011a). CC was more strongly correlated with the caregiver‐reported measure of imitation skills than YDI. This finding may be due to the fact that CC measures spontaneous imitation, and caregivers may be more likely recognize and/or report on instances of spontaneous imitation than elicited imitation or that CC may be more reflective of naturalistic interactions that parents engage in with their children.

Due to the focus of our study (social communication), we were limited in measures that could be examined to determine discriminant validity. Despite this limitation, we found evidence of discriminant validity; child demographic factors and caregiver‐reported behavior problems on the BITSEA, which has not been previously linked to imitation skills, were not correlated with performance on either imitation measure. It was also encouraging to see that imitation performance was not related to any caregiver demographics. This finding suggests that administration may be feasible for diverse families. Additional research may wish to examine discriminant validity with additional measures of child ability (e.g., gross motor skills).

Importantly, both measures predicted gains in Expressive Language as measured by the VABS over 9 months. This finding is consistent with prior studies (Frost et al., 2024; Pittet et al., 2022; Stone & Yoder, 2001), and provides support for the validity of the measures. It also adds to the growing literature showing the importance of imitation skills in predicting language growth in young autistic children. Interestingly, while concurrently associated with all measures of language, both YDI and CC were only associated with Expressive Language gains on the VABS over time. This is consistent with Frost et al., 2024, which found that imitation skills on the original UIA (CC) were predictive of expressive but not Receptive Language growth over 9 months in autistic children.

Both imitation measures were also sensitive to change over 4 months, suggesting that YDI and CC should be sensitive to treatment effects over relatively short durations. We limited our longitudinal analyses to children who were in the RIT later group, indicating that young children with social communication delays show improvement in imitation skills over a relatively short duration even in the absence of direct intervention targeting imitation. This is consistent with Pittet et al. (2022) who found significant improvement in imitation skills among many young autistic children over the preschool period.

There are several limitations that should be acknowledged. First, we did not conduct any in‐person assessments. Thus, we were unable to validate the virtual assessments against the original imitation measures or other standardized measures of child performance implemented by a trained examiner. Future work would benefit from further validation with examiner‐administered assessments. In addition, we did not have a comparison sample of neurotypical children or children with other developmental conditions. Thus, it is not clear how performance would compare between children with social communication delays and those without. Further, all children were under the age of 30 months when enrolled in the study; therefore, it is unknown how these measures might work with older children. Finally, the children in our study were referred for social communication delays; autism was not confirmed until the Time 3 assessment. So far, 89% of the sample who have completed diagnostic evaluation at the Time 3 assessment (*n* = 104) have received an autism diagnosis based on DSM‐5 criteria (supported by the VABS, caregiver interview, and a CARS rating based on the full CPP). However, future research with children with a confirmed diagnosis of autism may be important.

Taken together our results suggest that imitation skills in young children with early signs of autism can be measured effectively using a caregiver‐implemented assessment. This provides greater opportunity for virtual clinical trials targeting social communication in young children. Future work could explore the use of this approach by other individuals, such as community providers, either as the implementor or as the virtual assessor. Additional psychometric analysis of other virtual measures of social communication skills is needed.

## FUNDING INFORMATION

This work was supported by the National Institute of Mental Health (NIMH) of the National Institutes of Health (NIH) by Grant Numbers 1 R01MH122725‐01, 1 R01 MH122726‐01, 1 R01 MH122727‐01, and 1 R01 MH122728‐01. The content is solely the responsibility of the authors and does not necessarily represent the official views of the NIH.

## CONFLICT OF INTEREST STATEMENT

The authors declare no conflicts of interest.

## ETHICS STATEMENT

This study conformed to the standards of the U.S. Federal Policy for the Protection of Human Subjects and was approved by Michigan State University's Institutional Review Board.

## CONSENT

All parent/legal guardians who participated in this study provided informed written consent.

## Data Availability

Data that support this study are available upon request from the corresponding author.

## Footnotes

^1^
For a detailed description of our remote assessment protocol, please see Tagavi et al. (2024).

^2^
We only examined longitudinal data for the RIT later group to avoid premature analysis of outcome data in the clinical trial.
